# Dispersive optical activity for spectro-polarimetric imaging

**DOI:** 10.1038/s41377-025-01766-5

**Published:** 2025-02-20

**Authors:** Zhijie Cao, Siwei Sun, Jingxuan Wei, Yong Liu

**Affiliations:** School of Optoelectronic Science and Engineering, University of Electronic Science and Technology of China, Chengdu, 611731 China

**Keywords:** Spectrophotometry, Imaging and sensing

## Abstract

A spectro-polarimetric imaging approach leverages optical rotatory dispersion in natural crystals to encode spectral information into polarization states. The system demonstrates effectiveness in laboratory and outdoor field experiments, showing potential for biological microscopy, machine vision, and remote sensing applications.

Optical spectroscopy and spectrometric imaging have established themselves as indispensable analytical tools in modern science and technology. Their applications span diverse fields, from astronomical observation and industrial manufacturing to environmental monitoring and medical diagnostics. At its core, spectral extraction represents a high-dimensional vector reconstruction problem, requiring two fundamental operations: the construction of appropriate high-dimensional basis vectors to represent the spectral space, and the implementation of inverse algorithms to reconstruct the original spectral information from measured projections^1^. Contemporary spectral extraction technologies primarily employ three approaches: dispersive gratings, narrowband filters, and Fourier transform spectroscopy. These methods share a common mathematical foundation based on quasi-orthogonal basis vectors, enabling relatively straightforward reconstruction algorithms. In dispersive gratings and narrowband filters, optical spectra are decomposed into delta-function-like basis vectors centered at distinct wavelengths with minimal overlap. Fourier transform spectroscopy employs basis vectors with periodic spectral responses, determined by either mirror displacement or interferometer voltage^2,3^, utilizing the inherent orthogonality of sinusoidal functions. While traditional approaches rely on orthogonal basis vectors, this requirement poses significant challenges for device miniaturization. A paradigm shift is emerging in spectroscopic technology, and the key insight driving the innovation is that strict orthogonality is not fundamentally necessary for spectral reconstruction—linear independence of basis vector suffices. This realization has given rise to computational spectroscopy, which deliberately employs non-orthogonal spectral functions as reconstruction bases. Although this approach demands more sophisticated reconstruction algorithms, often involving machine learning techniques, it substantially relaxes device fabrication constraints and enables unprecedented miniaturization possibilities^4^.

The foundation of computational spectroscopy rests on the design of non-orthogonal spectral response functions, which characterize how optical fields respond to different wavelengths. This wavelength discrimination primarily relies on two fundamental mechanisms: material dispersion and structural dispersion. Material dispersion emerges from wavelength-dependent refractive indices, manifesting in both real and imaginary components. The imaginary component, responsible for wavelength-selective absorption, has been successfully demonstrated in recent breakthrough applications^5–7^. The real component, governing phase differences, typically requires specific optical architectures like prisms to achieve effective spectral separation. Structural dispersion, conversely, arises from the interaction between light and engineered architectures. In structures such as Fabry–Pérot cavities^8^ and metasurface^9,10^, different wavelengths generate distinct propagation phases, exciting various resonant modes that manifest as measurable intensity variations. While current techniques primarily convert wavelength information into intensity measurements, light possesses additional dimensions for exploitation—including polarization, momentum, and orbital angular momentum. This multidimensional character of light suggests the possibility of a new paradigm: high-dimensional dispersion that correlates spectral information with other optical properties, potentially revolutionizing spectroscopic capabilities^11^.

Now, writing in *eLight*, Wang et al. from Purdue University and The University of New South Wales proposed an innovative computational spectroscopy that employs dispersive optically active crystals, such as α-quartz, to achieve spectral separation in the polarization dimension rather than the conventional spatial dimension^12^. This method exploits the wavelength-dependent optical rotation characteristics of these crystals, where different wavelengths undergo distinct polarization rotations, effectively encoding spectral information into polarization states. In their practical implementation, the team designed a basic unit consisting of five key components: a fixed input polarizer, an optically active crystal, a mechanically rotating polarizer, another optically active crystal with opposite handedness, and a fixed output polarizer with the same direction as the input polarizer. This arrangement provides two practical advantages: it simplifies operation by requiring rotation of only one polarizer, and aligns the output polarization with the input, enabling efficient cascade connection of multiple units. Additionally, rotating the entire assembly enables control of polarized light entering the imaging system, achieving polarization state detection similar to traditional time-division polarization imaging schemes using rotating polarizers. Combined with a conventional monochrome camera, this creates the compact and efficient Nonlocal-Cam spectro-polarimetric imaging system (Fig. 1a). Then, the system employs a “learning-sampling-reconstruction” approach incorporating compressed sensing and dictionary learning algorithms, reducing computational and training data requirements compared to traditional methods. The team validated this system through three stages of testing. Basic spectral imaging tests confirmed accurate spectral reconstruction across multiple pixels. Polarization imaging of plastic eyewear demonstrated the ability to capture wavelength-dependent stress-induced birefringence. Outdoor field testing achieved spectro-polarimetric imaging of natural scenes, revealing distinct polarization characteristics between artificial objects like vehicles and natural features such as vegetation. These tests confirmed the successful acquisition of four-dimensional data incorporating spatial, spectral, and polarimetric information.

**Fig. 1:**
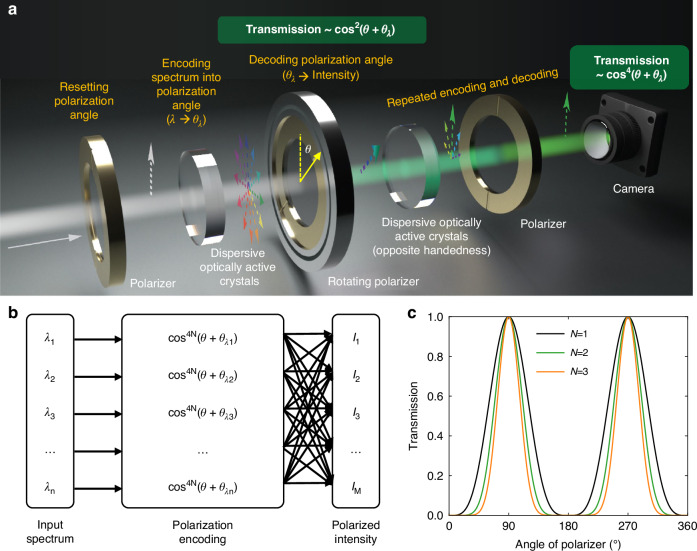
**Operating principle of the spectro-polarimetric imaging system.** **a** Schematic of the spectro-polarimetric camera based on optically active materials. **b** System diagram showing the mapping between input wavelengths (*λ*_1_ to *λ*_*n*_) and measured polarized intensities (*I*_1_ to *I*_*M*_) through polarization encoding functions. *θ*_*λn*_ is the rotated polarization for a specific wavelength of *λ*_*n*_. **c** Normalized transmission curves for different unit numbers (*N* = 1, 2, 3) of the polarization encoding function versus polarizer angle, demonstrating how increasing *N* sharpens the response

At first glance, leveraging polarization dimensions for spectral encoding might seem limiting to the optics community, given that linearly polarized light is fully characterized by just three parameters: intensity, polarization angle, and degree of polarization (equivalently expressed through Stokes parameters *S*_0_, *S*_1_, and *S*_2_)—suggesting a constrained measurement basis. This apparent constraint has a precise mathematical expression: a measurement vector sampled at different polarization angles *θ* follows the form *A*·cos^2^(*θ* + *θ*_0_), where *A* and *θ*_0_ are arbitrary constants. Any matrix constructed from such vectors has a maximum rank of 3. Consequently, the complete polarization state can be decomposed into a linear combination of just three basis vectors cos^2^(*θ* + *θ*_*i*_) where *i* = 1, 2, 3. This mathematical framework validates the traditional understanding that three measurements through a rotating analyzer suffice to characterize linearly polarized light.

However, in a unit designed as in this work, the incident light is rotated and polarized twice, so the output light would follow the form *A*·cos^4^(*θ* + *θ*_*λ*_), where *θ*_*λ*_ is the wavelength-dependent angle (Fig. 1b). It can be mathematically proven that such an expression will require at least five basis vectors for complete linear combination. That is, each basic unit (polarizer, left-handed crystal, adjustable polarizer, right-handed crystal, polarizer) creates a five-dimensional polarization space for spectral encoding. The preservation of polarization alignment between output and input states enables efficient cascade connection of basic units. As unit count (*N*) increases, the output spectral function evolves as cos^4*N*^(*θ* + *θ*_*λ*_), yielding 4*N* + 1 degrees of freedom. This progression enhances both spectral information storage capacity and resolution, which manifests as enhanced spectral response sharpness (Fig. 1c). The experimental protocol utilized measurements at 100 distinct polarizer angles, surpassing the inherent degrees of freedom of the one-unit imaging system of 5. While exceeding the inherent degrees of freedom of the system, this oversampling strategy serves a crucial practical purpose for signal processing: it enhances the signal-to-noise ratio through averaging and strengthens the robustness of spectral reconstruction.

Notably, although optically activity and its dispersion have been long recognized^13^, the underlying physics of its nonlocal light-matter interaction and associated dispersion phenomena have only recently emerged as subjects of rigorous academic investigation^14^. This work establishes comprehensive theoretical foundations through precise derivations of optical activity Lagrangian and energy density expressions. These formulations illuminate the fundamental physics of nonlocal dispersion while revealing intrinsic relationships among optical rotation, photonic spin density, and optical chirality density. A distinctive feature of this nonlocal dispersion manifests as “super-dispersion,” wherein optical rotatory power exhibits inverse dependence on *λ*^2^. While this relationship has been documented in classical literature^13^, the theoretical framework provides novel insights that advance the development of spectral imaging systems based on optically active materials.

In summary, the Nonlocal-Cam system demonstrates compelling practical advantages through its use of readily available natural crystals, circumventing the need for complex nanofabrication processes and precise control mechanisms. Its compact architecture enables potential integration with mobile platforms such as drones, while offering tunable spectral resolution through adjustable filtering units. Importantly, the operational range extends beyond conventional liquid crystal technologies—typically confined to visible and near-infrared regions by ITO electrode limitations—by utilizing TeO_2_ and Te crystals for short-wave and mid-wave infrared applications, respectively. These characteristics position the technology for diverse applications across critical domains, including biological microscopy, physics-driven machine vision, remote sensing, and space exploration, offering new possibilities for technological innovation in these fields.

Meantime, several engineering challenges remain to be addressed. The current implementation requires ~10 s for spectral reconstruction due to time-division measurements and mechanical operations. Light throughput is constrained by the non-perfect transmission of quartz crystal (~90%) and polarizers (~85%), which will be further constrained when cascading multiple units. The required crystal thickness of 6 mm/unit, driven by weak circular birefringence in natural materials, might be reduced through artificially engineered chiral metasurface^15^. Alternatively, anisotropic materials present promising opportunities, as demonstrated by the birefringence in calcite of 0.172—three orders of magnitude greater than the circular birefringence in quartz. However, these materials operate through distinct polarization mechanisms: optically active crystals modulate polarization along the latitude of the Poincaré sphere, requiring linear polarizer filtering, whereas anisotropic materials modulate along the longitude, necessitating circular polarization filtering (typically implemented using a combination of linear polarizers and quarter-wave plates). Moreover, the current implementation is limited to processing linearly polarized light, lacking the capability to measure circular polarization components (Stokes parameter *S*_3_) of incident light—a critical direction for future research advancement^16^.

Looking forward, breakthrough opportunities in multidimensional optical field sensing may emerge through establishing ingenious correlations between different parameter spaces. The approach of spectral sensing through dispersive polarization optical elements represents an inspirational paradigm: establishing spectral-polarization-intensity information mapping chains and expanding information capacity through cascaded units. This cross-dimensional information encoding methodology promises to inspire innovative developments in optical field manipulation and sensing.
